# Bacteria in Crude Oil Survived Autoclaving and Stimulated Differentially by Exogenous Bacteria

**DOI:** 10.1371/journal.pone.0040842

**Published:** 2012-09-17

**Authors:** Xiao-Cui Gong, Ze-Shen Liu, Peng Guo, Chang-Qiao Chi, Jian Chen, Xing-Biao Wang, Yue-Qin Tang, Xiao-Lei Wu, Chun-Zhong Liu

**Affiliations:** Department of Energy and Resources Engineering, College of Engineering, Peking University, Beijing, P.R. China; Loyola University Medical Center, United States of America

## Abstract

Autoclaving of crude oil is often used to evaluate the hydrocarbon-degrading abilities of bacteria. This may be potentially useful for bioaugmentation and microbial enhanced oil recovery (MEOR). However, it is not entirely clear if “endogenous” bacteria (e.g., spores) in/on crude oil survive the autoclaving process, or influence subsequent evaluation of the hydrocarbon-degradation abilities of the “exogenous” bacterial strains. To test this, we inoculated autoclaved crude oil medium with six exogenous bacterial strains (three *Dietzia* strains, two *Acinetobacter* strains, and one *Pseudomonas* strain). The survival of the spore-forming *Bacillus* and *Paenibacillus* and the non-spore-forming mesophilic *Pseudomonas, Dietzia, Alcaligenes*, and *Microbacterium* was detected using a 16S rRNA gene clone library and terminal restriction fragment length polymorphism (T-RFLP) analysis. However, neither bacteria nor bacterial activity was detected in three controls consisting of non-inoculated autoclaved crude oil medium. These results suggest that detection of endogenous bacteria was stimulated by the six inoculated strains. In addition, inoculation with *Acinetobacter* spp. stimulated detection of *Bacillus*, while inoculation with *Dietzia* spp. and *Pseudomonas* sp. stimulated the detection of more *Pseudomonas*. In contrast, similar exogenous bacteria stimulated similar endogenous bacteria at the genus level. Based on these results, special emphasis should be applied to evaluate the influence of bacteria capable of surviving autoclaving on the hydrocarbon-degrading abilities of exogenous bacteria, in particular, with regard to bioaugmentation and MEOR. Bioaugmentation and MEOR technologies could then be developed to more accurately direct the growth of specific endogenous bacteria that may then improve the efficiency of treatment or recovery of crude oil.

## Introduction

Crude oil is one of the most important industrial resources and energy sources in modern society. Since the depletion of easily recoverable crude oil deposits, microbial enhanced oil recovery (MEOR) has gained increasing interest because it is environment friendly and cost efficient [1]. At the same time, oil spills from the exploitation, transportation, and utilization of crude oil pose a risk to soil, aquifer, and groundwater environments [2]. Among clean-up technologies, bioremediation is the optimal method applied worldwide as it is both environment friendly and economical in nature. Bioaugmentation and “exogenous” MEOR technologies involve the introduction of “exogenous” microorganisms into oil reservoirs and contaminated environments to increase the efficiency of petroleum degradation, improve oil recovery, and extend the life of oil wells [1], [3], [4]. The selection of optimal exogenous microorganisms is therefore very important for successful exogenous MEOR and bioaugmentation.

In general, the selection of effective microorganisms is often made using previously autoclaved cultures with crude oil. However, recent studies reported the presence of various bacteria in crude oil, which were detected by both culture-independent and culture-dependent methods after the crude oil was isooctane pretreated. For example, mesophilic *Ochrobactrum, Burkholderia, Stenotrophomonas, Brevundimonas, Pseudomonas*, *Bacillus*, *Sphingobacterium, Acinetobacter*, *Propionibacterium*, *Sphingobium,* and *Bacillales* were detected in crude oil from the Middle East and Japan [5], [6], while the relatives of the thermophilic *Petrotoga*, *Clostridium*, and *Synergistetes* were more frequently detected in Chinese and Japanese oil samples [5]. The presence of microorganisms in crude oil evokes several questions. First, do the bacteria, perhaps in the form of spores, survive autoclaving? Second, do the surviving bacteria become active and exert their influence on crude oil degradation? Third, does this bacterial activity confound the accurate evaluation of the abilities of the inoculated “exogenous” bacterium? Finally, if the bacteria in the crude oil become active, how does the inoculated “exogenous” bacterium respond? Answers to these questions would be crucially important to accurately evaluate the abilities of exogenous microorganisms for the successful application of MEOR and bioaugmentation. To date, no study has effectively addressed these questions.

Therefore, we conducted an investigation on the growth and survival of both “endogenous” and “exogenous” bacteria in autoclaved crude oil. Our study found that the mesophilic, non-spore-forming “endogenous” bacteria in the crude oil samples survived the autoclaving process and were differentially stimulated by different inoculated “exogenous” bacteria. Surprisingly, these inoculated “exogenous” bacterial strains were then routinely out-competed by the “endogenous” bacteria.

## Materials and Methods

### Crude oil and bacterial strains

“Fresh” crude oil was sampled from the No. 3 Oil Product at the Daqing Oilfield, the largest oil field in China. The production liquid, which consists of both crude oil from the oil reservoir and injection water, was allowed to settle for one day in a sedimentation tank. Floating oil was then separated and dewatered by electrical dehydration for 40 min. Soon after dewatering, the crude oil was aseptically sampled for further experiments. The properties of the crude oil samples were as follows: density, 844.1 kg·m^−3^; viscosity, 23.34 mPa·s; solidification temperature, 32°C; and water content, 0.16%. The four sub-fraction contents (wt/wt) were saturated hydrocarbon (SH), 64.08%; aromatic hydrocarbon (AH), 18.91%; non-hydrocarbon (NH), 15.40%; and resins and sphaltenes (SP), 1.61%. In addition, the depth and the bottom hole temperature of the production well were 1140 m and 45°C, respectively.

Six bacterial strains, isolated from the production liquid from the Daqing Oilfield [7] and the oil-polluted saline soil at the Shengli Oilfield in eastern China, were selected for use because of their excellent ability in degrading hydrocarbons (Table 1). They were *Acinetobacter* sp. SLG510A3-32 (with the 16S rRNA gene sequence similarity of 99.6% to *Acinetobacter junii*), *Acinetobacter* sp. SLG310A2-8A1 (100.0% similar to *Acinetobacter venetianus*), *Pseudomonas* sp. SLG310A2-4A2 (98.2% similar to *Pseudomonas alcaliphila*), *Dietzia* sp. SLG510A3-3B2-2 (98.6% similar to *Dietzia maris*), *Dietzia* sp. DQ12-45-1b (99.4% similar to *Dietzia cercidiphylli*), and *Dietzia* sp. SLG510A3-30A2 (100.0% similar to *Dietzia maris*).

**Table 1:** Bacterial strains and cultures.

Culture	Inoculum strain		Degradation of crude oil component				
	Name	Phylogenetically closest relative	Paraffin	Crude oil	Tetracosane	Hexatriacontane	Aromatic compound
AJ-1	SLG510A3-32	*Acinetobacter junii*, 99.85%	+++	+++	++	(+)	Fluorene, +
DM-2	SLG510A3-3B2-2	*Dietzia maris*, 99.58%	++	+	−	−	Chrysene, +
DP-6	DQ12-45-1b	*Dietzia cercidiphylli,* 99.36	++	+	+	+	Phenothiazine,+; phenanthrene, +; Indole, (+); Fluorene,(+);Chrysene, (+)
AV-11	SLG310A2-8A1	*Acinetobacter venetianus*, 100.00%	+	+	+	+++	phenanthrene (+)
PA-13	SLG310A2-4A2	*Pseudomonas alcaliphila,* 98.19%	−	(+)	++	(+)	Phenol, ++
DM-24	SLG510A3-30A2	*Dietzia maris,* 100.00%	++	+++	+	(+)	Indole(+)

Note, The degradation of different crude oil components is represented by the symbols −, (+), +, ++, and +++ which stands for no, weak, normal, strong and very strong degradation.

### Cultivation of the crude oil bacterial community

Each of the six strains were pre-cultured in artificial sea water (ASW) medium [8] at 37°C for three days, harvested from 40 ml of culture medium by centrifugation at 8,000 rpm for 10 min, washed twice with minimal salt medium (MSM), and re-suspended in MSM to prepare the inoculum suspension. The composition of MSM was as follows (per liter): NaCl, 5 g; NH_4_H_2_PO_4_, 1 g; (NH_4_)_2_SO_4_, 1 g; MgSO_4_.7H_2_O, 0.2 g; KNO_3_, 3 g; K_2_HPO_4_, 1 g; pH 7.0) [2]. Two grams of the crude oil was added to 100 ml of MSM in a 250-ml flask and autoclaved at 121°C for 20 min to prepare the crude oil medium. The six inoculant cell suspensions were added to crude oil medium in six separate flasks with a final cell concentration of 2.89±0.2×10^6^ CFU·l^−1^. The six cultures were labeled as AJ-1, DM-2, DP-6, AV-11, PA-13, and DM-24 corresponding to the inoculated bacteria, namely, *Acinetobacter* sp. SLG510A3-32, *Dietzia* sp. SLG510A3-3B2-2, *Dietzia* sp. DQ12-45-1b, *Acinetobacter* sp. SLG310A2-8A1, *Pseudomonas* sp. SLG310A2-4A2, and *Dietzia* sp. SLG510A3-30A2, respectively. Three additional flasks containing only the crude oil medium without bacterial inoculum were used as controls. The flasks were incubated at 37°C in the dark and shaken at a speed of 150 rpm. Crude oil floating on the surface of the inoculated cultures gradually dispersed during the incubation period, turning the medium black after the inoculation period. An increase in the turbidity of the inoculated cultures was also observed, suggesting the development of bacteria capable of utilizing crude oil for growth. In contrast, no visible changes were observed in the three control flasks. At days 4, 10, 20, 30, and 55, the inoculated cultures and non-inoculated controls were sacrificed for sampling. A well-mixed 20-ml portion of each 100-ml culture was centrifuged at 8,000 rpm for 10 min. The precipitated pellet containing bacterial cells was used for DNA extraction and bacterial community analysis. The remaining 80-ml portion of each culture was used to analyze the surface tension, pH, and residual oil components.

### Observation of microbial cells in crude oil

Crude oil samples, both un-autoclaved original samples and the autoclaved ones, were mixed well in the filtrate-sterilized kerosene and water solution [kerosene∶water, 9∶1 (v/v)] and viewed under a light microscope (XSZ-H3, COIC, Chongqing, China). Simultaneously, the crude oil-kerosene-water mixture was stained with 4′, 6-diamidino-2-phenylindole (DAPI), and then viewed with a fluorescence microscope (DM6000M, Leica, Germany) to check for the presence of microbial cells in the crude oil samples [9].

### DNA extraction and amplification

DNA from the cell pellets described above (six inoculated and three un-inoculated cultures) was extracted with a FastDNA® SPIN Kit for Soil (MP Biomedicals, LLC, California, USA), according to the manufacturer's instructions. DNA extraction from the original and the autoclaved crude oil samples was performed using a method modified from Yamane et al [5]. Briefly, 100 g of both the original and autoclaved crude oil samples were thoroughly mixed with 500 ml of *n*-hexane by vortexing, and centrifuged at 8000 rpm and 4°C for 20 min. After discarding the upper layer, the remaining mixture was filtered through a 0.22-µm polytetrafluoroethylene (PTFE) membrane filter to collect the cells. The filter was then cut into small pieces with sterilized scissors for DNA extraction with the FastDNA® SPIN Kit for Soil, according to the manufacturer's instructions. Bacterial 16S rRNA gene fragments were amplified from 50 ng of the DNA extract template by using the universal bacterial primer set, 8F (5′-AGAGTTTGATCCTGGCTCAG-3′) and 1492R (5′-GGTTACCTTGTTACGACTT-3′) in a PCR reaction performed with Taq™ DNA polymerase (TaKaRa Biotechnology; Dalian, China), according to the manufacturer's instructions. For terminal restriction fragment length polymorphism (T-RFLP) analysis, the 8F primer was labeled with 6-carboxyfluorescein (6-FAM) at the 5′ end. The PCR was performed with an initial denaturation at 95°C for 5 min; followed by 29 cycles of 1 min at 94°C, 45 s at 50°C, and 90 s at 72°C; and a final extension at 72°C for 10 min. The PCR amplicons were purified using a QIA Quick® PCR Purification Kit (QIAGEN GmbH, D-40724 Hilden, Germany), followed by T-RFLP analysis and clone library construction.

### T-RFLP analysis

T-RFLP analysis was performed according to a previously described protocol [10]. Briefly, 10 µl of a purified DNA amplicon (obtained with the 6-FAM labeled primer) was digested in a 20-µl reaction mixture for 6 h at 37°C with 20 U of *Hha* I (New England Biolabs, Beverly, MA, USA), according to the manufacturer's instructions. After a desalting step, the purified digested DNA was mixed with 12 µl of Hi-Di formamide and 0.5 µl of a DNA fragment length internal standard (GeneScan Liz-500; Applied Biosystem, IL, USA), denaturated at 95°C for 5 min, and immediately snap-cooled on ice. The “Genescan” analysis was then conducted in a capillary electrophoresis system (ABI 3130 Genetic Analyzer, Applied Biosystems), according to the manufacturer's instructions. Fragment separation data were collected with ABI 3130 Collection (version 2.7) and GeneMapper Analysis Software (version 3.7). The relative peak area of every single T-RF was calculated by dividing the individual T-RF peak area by the total area of all the peaks. Peaks with a terminal restriction fragment (T-RF) of 50–800 bp were regarded “effective.”

### Clone library analysis

As no DNA was recovered (confirmed by gel electrophoresis, UV spectrometry, and PCR) from the autoclaved crude oil samples or the controls, seven clone libraries were constructed for the amplified 16S rRNA gene fragments from the DNA retrieved from the original crude oil and from the six cultures that had been incubated for 55 days. Clone libraries were constructed, and the clones were analyzed, screened, and classified into groups as previously described [11], [12]. Representative clones from each group were randomly picked and sequenced. All the sequences were checked with the CHECK-CHIMERA program in the Ribosomal Database Project (RDP-II, http://wdcm.nig.ac.jp/RDP/html/analyses.html) [13]. Phylogenetic trees were then constructed using the neighbor-joining algorithm with the Jukes–Cantor correction factor in ARB [14]. Tree topology was also evaluated using the maximum parsimony and maximum-likelihood algorithms in ARB. Sequences with >97% similarity were defined as having the same OTU in the tree. All the sequences from the seven bacterial libraries were analyzed using the SILVA web server [http://www.arbsilva. de/; 15] and the MOTHUR [16] and LIBSHUFF programs [http://libshuff.mib.uga.edu/; 17] to determine the similarities and differences between the clone libraries. OTUs were determined at 97% sequence similarity. All bacterial libraries were clustered using the UPGMA algorithm based on the distance between communities, as calculated using the Yue-Clayton index [18]. A heatmap analysis using the Yue-Clayton index was also conducted to identify the pairwise similarities in the libraries.

### Chemical analyses

The surface tension of the culture samples was measured with a Kruss tensiometer (JJ2000B; Zhongchen, Shanghai, China) and reported as mN·m^−1^ at room temperature. pH values were measured with a pH meter. Analyses of the four subfractions of the residual oil were carried out as follows. Briefly, 80 ml of each culture sample was extracted twice with 80 ml of *n*-hexane, after which the oil was collected by centrifugation at 14,000 rpm for 10 min to remove water from the mixture. Samples were then air-dried in a fume hood overnight [12]. The extracted oil was fractionated into saturates, aromatics, non-hydrocarbons, and resins and asphaltenes by liquid-solid chromatography [19]. The fractions were weighed and calculated as ratios to the total petroleum hydrocarbon (TPH) content. The saturate and aromatic fractions were analyzed using gas chromatography coupled with mass spectrometry GC-MS (Thermo-Finnigan Trace-DSQ, TX, USA) with the HP-5MS column (60 m×0.2 mm×0.25 µm) and helium as carrier gas at a flow rate of 1 ml·min^−1^. The temperature of the injector and the detector was 300°C and 280°C, respectively. The temperature program for the analysis of the saturate fraction was as follows: 50°C for 1 min, increase by 20°C·min^−1^ to 120°C, increase by 4°C·min^−1^ to 250°C, increase by 3°C·min^−1^ to 310°C, hold for 30 min. The temperature program for the analysis of the aromatic fraction was as follows: 50°C for 1 min, increase by 15°C·min^−1^ to 120°C, increase by 3°C·min^−1^ to 300°C, hold for 35 min [10]. The chromatograms were then analyzed for the relative amount of different crude oil components. Diagnostic parameters such as the ratio of pristane to phytane (Pr/Ph) and the ∑C21−/∑C22+ ratio, which can be used to indicate the biological degradation of hydrocarbons [20], were then calculated.

## Results

### Functional properties of the crude oil bacterial community

**Figure 1:**
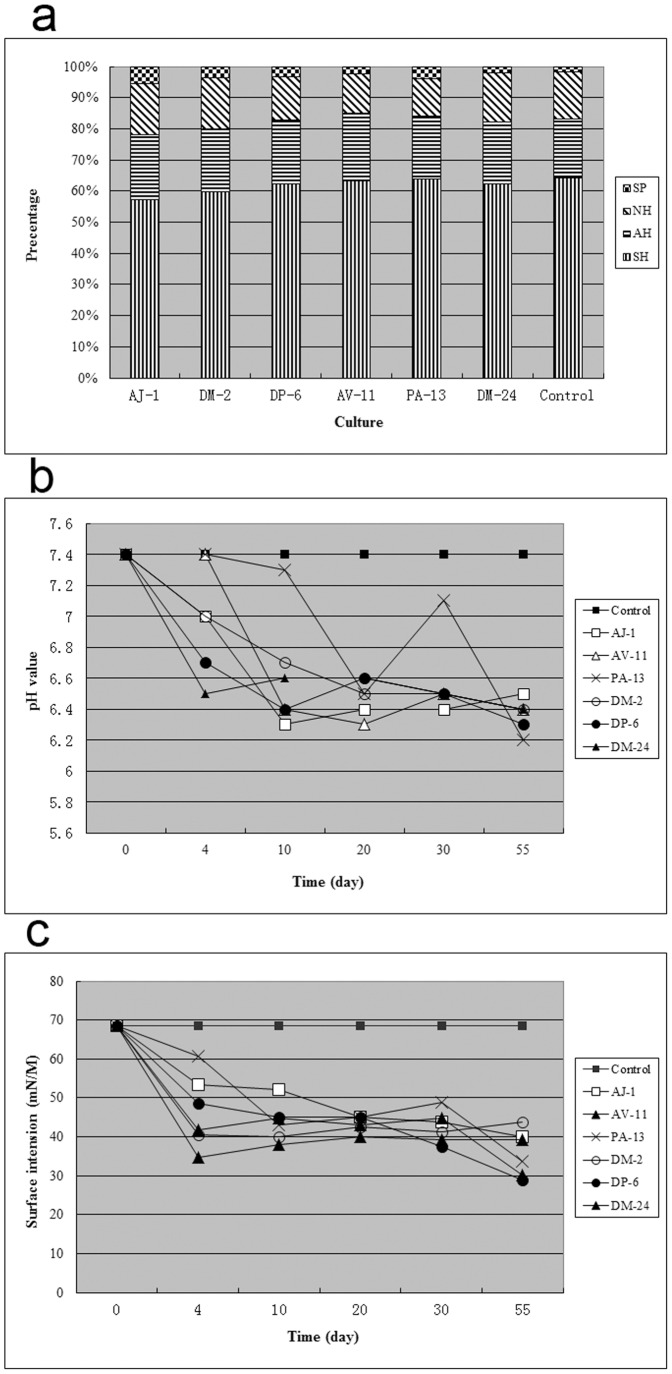
Functional properties of the crude oil cultures. (a) Composition of four oil subfractions, namely, SH, AH, NH, and SP, stands for the saturated hydrocarbon, aromatic hydrocarbon, non-hydrocarbon resin, and sphaltene parts of the crude oil; (b) change in pH; and (c) change in the surface tension.

Compared to the controls, the SH fraction decreased in all the cultures, the NH fraction decreased in the cultures DP-6, AV-11, and PA-13, while the ratios of AH and SP increased in most cultures (Figure 1a), suggesting different patterns of degradation. pH and surface tension values quickly decreased in all the inoculated cultures and were at low levels until the end of the experiment (Figure 1b, 1c). In addition, highly significant changes in the diagnostic parameters (Table 2) also directly supported microbial TPH degradation in the inoculated cultures. In contrast, no changes in the oil subfractions, pH values, or surface tension values were detected in the three controls, suggesting that the bacteria did not grow or perform degradation in the crude oil.

**Table 2:** Change of biodegradation indicators.

Diagnostic Parameters	Culture						
	Control	AJ-1	DM-2	DP-6	AV-11	PA-13	DM-24
∑C21−/∑C22+	1.16	0.68	1.09	0.58	0.79	0.63	0.85
Pr/Ph	1.30	1.12	1.16	1.15	1.19	1.20	1.13
Pr/nC17	0.17	0.39	0.30	0.52	0.30	0.46	0.35
Ph/nC18	0.14	0.30	0.26	0.35	0.23	0.31	0.28

Note: Pr and Ph stand pristane and phytane. ∑C21−/∑C22+ stands for the ratio of the sum of all the alkanes with chain length shorter than 21 carbon atoms to the sum of all the alkanes with chain length greater than 21 carbon atoms.

### Temporal change in the bacterial community

The non-autoclaved original crude oil was mixed with a filter-sterilized kerosene/water solution before the cell movement and DAPI signals were observed under light and fluorescence microscopy, respectively. A number of cell-like dots and movement were observed when the non-autoclaved samples were studied, whereas very few cell-like dots were observed in the autoclaved crude oil samples.

**Figure 2:**
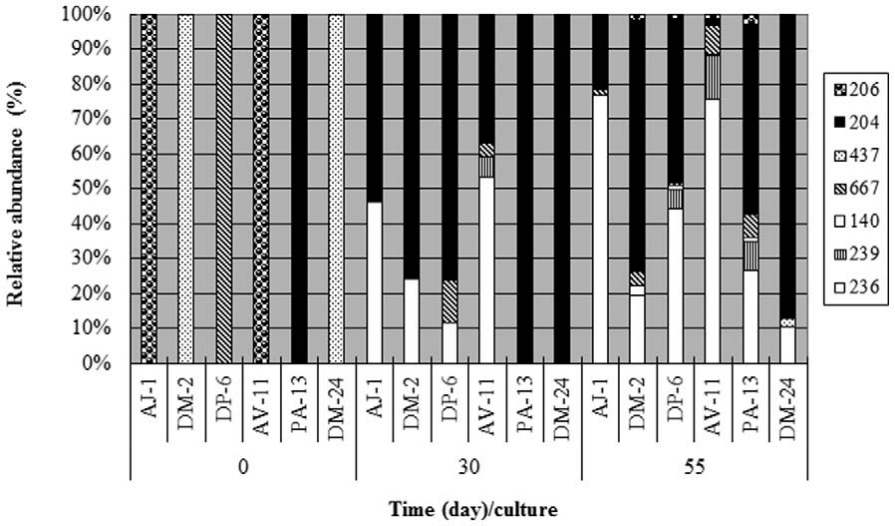
Change of relative abundance of different bacterial T-RFs.

At the beginning of the experiments and soon after the exogenous bacteria were inoculated, only one peak of T-RF could be detected in the inoculated culture. At day 30, the detectable bacteria in AJ-1 were those with T-RFs of 204 bp (*Pseudomonas* relatives) and 236 bp (*Bacillus* relatives). The predominant bacteria in DM-2 were also *Pseudomonas* (204 bp) and *Bacillus* (236 bp) relatives. Although *Pseudomonas* bacteria (T-RF 204 bp) were predominant in DP-6, the inoculated *Dietzia* strain as well as bacteria with a T-RF of 140 bp could also be detected. *Pseudomonas* (204 bp) and *Bacillus* (236 bp) relatives were again predominant in AV-11; however, bacteria with a T-RF of 239 bp could also be detected. In PA-13 and DM-24, only *Pseudomonas* relatives (204 bp) were detected. At day 55, the bacterial community became more diverse, and more T-RFs with higher evenness were detected. Interestingly, the abundance of *Bacillus* relatives (236 bp) increased, whereas that of *Pseudomonas* relatives (204 bp) decreased (Figure 2).

### Bacterial community revealed by clone library analysis

Although many attempts were made to collect bacterial cells by centrifugation, to extract DNA, and to amplify DNA from the autoclaved crude oil and the control medium samples, no success was obtained. Consequently, clone libraries could not be constructed for the microorganisms that may have been present in the autoclaved crude oil or control mixtures. Clone libraries were constructed only for the DNA retrieved from the original (not autoclaved) crude oil sample and from the six inoculated cultures at day 55 (The nucleotide sequences in this study were deposited in GenBank with the accession number of JN882027–JN882180).

The crude oil had astonishingly high bacterial diversity (Figure S1). The 53 clones represented 15 known genera and five phyla, namely, *Proteobacteria* (64.2%), *Firmicutes* (13.2%), *Actinobacteria* (5.7%), *Bacteroidetes* (7.6%), and *Deinococcus-Thermus* (7.6%), and an uncultured bacterial cluster (1.2%). The main bacterial clones belonged to *Acinetobacter* (30.2%), *Pseudomonas* (22.6%), *Bacillus* (9.4%), *Deinococcus* (7.6%), *Corynebacterium* (3.8%), and *Haloanella* (3.8%). *Bradyrhizobium*, *Methylophilus*, *Ralstonia*, *Massilia*, *Sphingomonas*, *Streptococcus*, *Rhodococcus*, *Exiguobacterium*, *Methylobacterium,* and Candidate Division TM7 accounted for 1.8% of the total clones each (Table 3). Clone sequences were about 98%–100% similar to database sequences with an exception of 93% similarity as noted below.

**Table 3:** Distribution of clones in crude oil clone library.

Classification	Alignment		Clones	
	Closest related bacterium	Similarity (%)	Number	Percentage (%)
**Phylum** *Proteobacteria*			44	64.2
**Class** *Alphaproteobacteria*			3	5.7
*Methylobacterium*	*Methylobacterium extorquens*	100.0	1	1.9
*Sphingomonas*	*Sphingomonas* sp. SKJH-30	99.9	1	1.9
*Bradyrhizobium*	*Bradyrhizobium japonicum*	100	1	1.9
**Class** *Betaproteobacteria*			3	5.7
*Methylophilus*	*Methylophilus methylotrophus*	99.3	1	1.9
*Massilia*	*Massilia* sp. PB248	97.9	1	1.9
*Ralstonia*	*Ralstonia* sp. AU378	99.9	1	1.9
**Class** *Gammaproteobacteria*			28	52.8
*Pseudomonas*	*Pseudomonas aeruginosa*	99.9–100	8	15.0
	*Pseudomonas gessardii*	99.9	2	3.8
	*Pseudomonas mandelii*	98.0	2	3.8
*Acinetobacter*	*Acinetobacter sp.* TK006	99.9–100	8	15.0
	*Acinetobacter* sp. TPR15	99.7–99.8	3	5.7
	Uncultured *Acinetobacter* clone 85	99.5–100.0	3	5.7
	*Acinetobacter junii*	98.2	1	1.9
	*Acinetobacter* sp. SD1	99.8	1	1.9
**Phylum** *Frimicutes*			7	13.2
*Bacillus*	*Bacillus mojavensis*	98.7–99.9	4	7.5
	*Bacillus simplex*	99.2	1	1.9
*Streptococcus*	*Streptococcus mitis*	99.3	1	1.9
*Exiguobacterium*	*Exiguobacterium mexicanum*	99.7	1	1.9
**Phylum** *Actinobacteria*			3	5.7
*Rhodococcus*	*Rhodococcus cercidiphyllus*	99.5	1	1.9
*Corynebacterium*	*Corynebacterium afermentans*	99.6–99.8%	2	3.8
**Phylum** *Bacteroidetes*			4	7.6
*Haloanella*	*Haloanella gallinarum*	98.3	2	3.9
*Unknown Bacteroidetes*	*Chryseobacterium* sp. pp2f,	93.1	1	1.9
Flavobacteriaceae	Flavobacteriaceae bacterium YMS-2	99.1	1	1.9
**Phylum** Deinococcus-Thermus			4	7.6
*Deinococcus*	*Deinococcus geothermalis*	99.5–100.0	4	7.5
**Others**			1	1.9
Uncultured bacterium	Candidate Division TM7 bacterium	97.7	1	1.9
Total			53	100.0

In the phylum *Proteobacteria*, *Acinetobacter* bacteria were closely related to *Acinetobacter* sp. TK006 (eight clones), *Acinetobacter* sp. TPR15 (three clones), uncultured *Acinetobacter* clone 85 (three clones), *A. junii* (one clone), and *Acinetobacter* sp. SD1 (one clone). The *Pseudomonas* bacteria were closely related to *Pseudomonas aeruginosa* (eight clones), *Pseudomonas gessardii* (two clones), and *Pseudomonas mandelii* (two clones). The other *Proteobacteria* clone sequences were closely related to *Methylophilus methylotrophus*, *Methylobacterium extorquens*, *Ralstonia* sp. AU378, *Massilia* sp. PB248, *Sphingomonas* sp. SKJH-30, and *Bradyrhizobium japonicum* with one clone each. Bacteria in the *Actinobacteria* phylum were closely related to *Rhodococcus cercidiphyllus* and *Corynebacterium afermentans* with one clone each. Bacteria in the *Firmicutes* phylum were closely related to *Bacillus mojavensis* (four clones), *Bacillus simplex* (one clone), *Streptococcus mitis* (one clone), and *Exiguobacterium mexicanum* (one clone). *Bacteroidetes* spp. were closely related to *Haloanella gallinarum* (two clones), *Chryseobacterium* sp. pp2f (one clone with sequence similarity of 93.1%), and *Flavobacterium* sp. YMS-2 (one clone).

Numerous mesophilic non-spore-forming bacteria other than the inoculated strains were unexpectedly detected in the clone libraries from inoculated cultures. The inoculated bacteria, on the other hand, were either not detected at all or had relatively low abundance (Figure S1, Table 4). In the clone library AJ-1, the predominant bacteria detected were *Bacillus* relatives, accounting for 69.4% of the 85 clones in the library. *Pseudomonas* relatives and *Dietzia* relatives (closely related to *Dietzia psychralcaliphila* with 16S rRNA sequence similarity of 99.9%) accounted for 28.2% and 1.2% of the clones, respectively. In the *Bacillus* group, 52 clones (58.8% of the total clones) and seven clones (9.4%) were closely related to *B. simplex* and *B. mojavensis* with sequence similarities of 96.3%–100.0%. The 16S rRNA sequence of 24 clones was 97.9%–100% similar to that of *P. pseudoalcaligenes*.

**Table 4:** Distribution of clones in the culture clone libraries.

Closely related bacteria	Library AJ-1		Library DM-2		Library DP-6		Library AV-11		Library PA-13		Library DM-24	
	Number/Ratio (%)	Similarity (%)	Number/Ratio (%)	Similarity (%)	Number/Ratio (%)	Similarity (%)	Number/Ratio (%)	Similarity (%)	Number/Ratio (%)	Similarity (%)	Number/Ratio (%)	Similarity (%)
**Phylum** *Frimicutes*	59 (69.4)		14 (18.9)		31 (35.6)		65 (85.5)		14 (21.2)		6 (6.5)	
**Genus** *Bacillus*	59 (69.4)		11 (14.7)		27 (31.0)							
*B. simplex*	52 (61.2)	96.9–99.7	-	-								
*B. mojavensis*	7 (8.2)	96.3–100.0			20 (23.0)	98.8–100.0	65 (85.5)	99.1–99.9	8 (12.1)	98.2–100.0		
*Unknown Frimicutes*			3 (4.2)	*B.niabensis* 90.0	1 (1.1)	*B.mojavensis* 94.6						
*B. niabensis*	-	-	11 (14.7)	98.9–99.1	7 (8.0)	98.6–99.0					6 (6.5)	99.3
**Genus** *Paenibacillus*	-	-	-	-								
*P. amylolyticus*	-	-	-	-	3 (3.4)	98.6–98.7			6 (9.1)	98.4–98.7		
**Phylum** *Proteobacteria*	25 (29.4)	-	57 (77.0)		51 (58.6)		2 (2.6)		49 (74.2)		84	
**Genus** *Alcaligenes*	-	-	1 (1.4)		1 (1.1)							
*A. faecalis*	-	-	1 (1.4)	99.8	1 (1.1)	99.8	2 (2.6)	99.5–99.7				
**Genus** *Pseudomonas*	24 (28.2)		40 (54.1)		50 (57.5)				48 (72.7)		67 (72.8)	
*P. pseudoalcaligenes*	24 (28.2)	97.9–99.7	40 (54.1)	99.4–99.7	49 (56.3)	99.3–99.7			48 (72.7)	99.4–99.7	67 (72.8)	99.0–99.6
*Unknown Proteobacteria*	1 (1.2)	*P. pseudoalcaligenes*, 94.4	16 (22.6)	*P. pseudoalcaligenes* 92.3–93.9					1 (1.5)	*P. anguilliseptica,* 94.5	17 (18.5)	*P. pseudoalcaligenes*, 90.2–91.0
*P. anguilliseptica*	-	-	-	-	2 (2.3)	97.5						
**Phylum** *Actinobacter*	1 (1.2)		2 (2.7)		2 (2.3)		8 (10.5)		3 (4.5)		2 (2.2)	
**Genus** *Dietzia*	1 (1.2)		2 (2.7)		-	-						
*D. psychralcaliphila*	1 (1.2)	99.9	2 (2.7)	99.5	-	-	6 (7.9)	98.3–99.5				
*D. maris*	-	-	-	-	-	-					2 (2.2)	99.9
*D. natronolimnaea*							1 (1.3)	95.7	3 (4.5)	99.7–99.9		
*Dietzia* sp. ES-QY-1	-	-	-	-	-	-	1 (1.3)	99.8				
**Genus** *Microbacterium*	-	-			2 (2.3)							
*M. testaceum*	-	-			2 (2.3)	99.6						
**Other**	-	-	1 (1.4)	Uncultured hydrocarbon contaminated clone TA8, 91.8	3 (3.4)	Bacterium N159B.20093.4%–97.5%	1 (1.3)	Bacterium N159B.200: 95.5%				
**Tatal**	85	100	74	100	87	100	76		66		92	

In the clone library DM-2, the predominant bacteria detected were *Pseudomonas* relatives, accounting for 54.1% of the total 74 clones, followed by relatives of an unassigned *Proteobacteria* (22.6%), *Bacillus* (18.9%), and *Dietzia* (2.7%). The *Pseudomonas*, *Bacillus,* and *Dietzia* relatives were most closely related to *P. pseudoalcaligenes, B. niabensis,* and *D. psychralcaliphila* with sequence similarities of 98.9%–99.8%. The remaining bacterial clones were closely related to *Alcaligenes faecalis* and the uncultured hydrocarbon contaminated clone TA8 with sequence similarities of 99.8% and 91.8%, respectively. The relatives of unassigned *Proteobacteria* and *Firmicutes* were closely related to *P. pseudoalcaligenes* (16 clones) and *B. niabensis* (3 clones) with sequence similarities of 92.3%–93.9% and 90.0%.

In the DP-6 library, *Pseudomonas* sequences dominated the clone library again, accounting for 57.5% of the 87 clones, followed by relatives of *Bacillus* (31.0%), *Paenibacillus* (3.4%), *Microbacterium* (2.3%), and *Alcaligenes* (1.1%). The *Pseudomonas* relatives included 49 clones that were closely related to *P. pseudoalcaligenes* and two clones that were related to *P. anguilliseptica*. The *Bacillus* relatives were closely related to *B. mojavensis* (21 clones) and *B. niabensis* (7 clones). The bacteria belonging to *Paenibacillus*, *Microbacterium*, and *Alcaligenes* were closely related to *P. amylolyticus*, *M. testaceum*, and *A. faecalis* with sequence similarities of 98.6%–99.8%. In addition, three clones were closely related to an uncultured bacterium, Bacterium N159B.200, with sequence similarities of 93.4%–97.5%.

In the AV-11 library, *Bacillus* bacteria were dominant, accounting for 86.8% of the 76 clones, followed by *Dietzia* (10.5%) and *Alcaligenes* (2.6%) relatives. The *Bacillus* relatives were closely related to *B. mojavensis* (65 clones) with sequence similarities of 99.1%–99.9%. The bacteria belonging to *Dietzia* were closely related to *D. psychralcaliphila* (6 clones) and *Dietzia* sp. ES-QY-1 (2 clones) with sequence similarities of 95.7%–99.8%. The bacteria belonging to *Alcaligenes* were closely related to *A. faecalis* with about 100% sequence similarity.

*Pseudomonas* bacteria were dominant in the PA-13 library, accounting for 74.2% of the total 66 clones in the clone library. Less dominant strains included *Bacillus* (12.1%), *Paenibacillus* (9.1%), and *Dietzia* (4.5%). *Pseudomonas* relatives included bacteria closely related to *P. pseudoalcaligenes* (48 clones) and *Pseudomonas anguilliseptica* (1 clone) with sequence similarities of about 100% and 94.5%, respectively. The *Bacillus, Paenibacillus,* and *Dietzia* relatives were closely related to *B. mojavensis, P. amylolyticus,* and *D. natronolimnaea* with sequence similarities of 98.2%–99.8%.

The inoculated *Dietzia* strain was detected in the DM-24 library, but with relatively low abundance. The dominant bacteria detected were again *Pseudomonas* (72.8% of the total 92 clones), followed by relatives of an unassigned *Proteobacteria* (18.5%), *Bacillus* (6.5%), and *Dietzia* (2.2%). Closely-related bacteria included *P. pseudoalcaligenes* (67 clones), *P. pseudoalcaligenes* (17 clones), *B. niabensis* (6 clones), and *D. maris* (2 clones) with sequence similarities of 99.0%–99.6%, 90.2%–91.0%, 99.3%, and 99.9% respectively.

In summary, the inoculated *Acinetobacter* sp., *Pseudomonas* sp., and *Dietzia* sp. in DM-2 and DP-6 could not be detected. In addition, different bacterial inoculates stimulated different microorganisms: the *Acinetobacter* inoculum stimulated the detection of higher number of *Bacillus* strains than *Pseudomonas*, while the *Dietzia* and *Pseudomonas* inoculants stimulated the detection of higher number of *Pseudomonas* strains than *Bacillus.* At lower taxonomic levels, differences in detection of bacterial species in the same genus were noted (Table 4). Among all the bacteria detected, only *Bacillus mojavensis* and *Bacillus niabensis* relatives were detected in the original crude oil and the inoculated crude oil samples. In contrast, the dominant bacteria detected in the inoculated cultures were not detected in the original crude oil.

### Comparisons among the clone libraries

**Figure 3:**
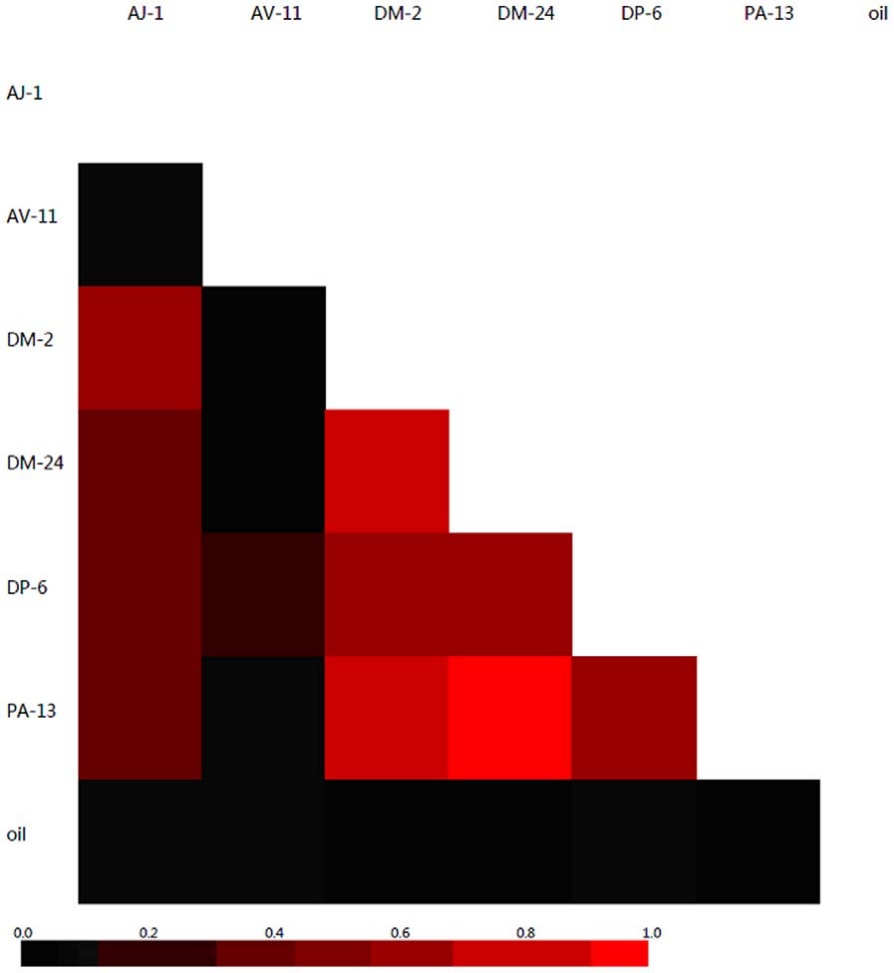
Heatmap analysis. Clones of the crude oil group and the six culture libraries were designated into OTUs defined at 97% in sequence similarity. The pairwise similarities among the seven groups were calculated using the Yue-Clayton index. In the heatmap, a lower triangle matrix was constructed with each color block showing the similarity in the community structure of two libraries. The similarity increases with the color, in the order of black to red.

UPGMA trees of the clone libraries revealed the similarity of the bacterial communities. The PA-13 and DM-24 libraries showed the maximum similarity. The PA-13, DM-24, DM-2, and DP-6 libraries formed a larger cluster and had a relatively high level of similarity in community structure. The AJ-1 and AV-11 libraries were further apart from the above four libraries. The oil library was left as an out-group (data not shown). Similar relationships were also expressed in the heatmap; the libraries from DM-2, DM-24, DP-6, and PA-13 were more similar to each other with brighter colors when compared to the other libraries (Figure 3). Together, these results support the idea that different exogenous bacteria stimulate different endogenous bacteria, while similar inoculants stimulate similar endogenous bacteria.

## Discussion

### Diverse mesophilic bacteria in a medium-temperature subterranean oil reservoir

Both thermophilic and mesophilic microorganisms were recently detected in oil reservoirs around the world [21]–[25]. Although the rarefraction curve of the clones did not reach saturation (Figure S2), analyses of the microbial DNAs retrieved from the chloroform-pretreated crude oil suggested that the bacterial community in the crude oil samples from Daqing Oilfield were much more diverse (Figure S1, Table 3). They included bacteria belonging to 15 genera from five known phyla, namely, *Firmicutes*, *Actinobacteria*, *Proteobacteria*, *Bacteroidetes,* and *Deinococcus-Thermus*, as well as an unknown cluster. Of the 15 genera, mesophilic *Acinetobacter* and *Pseudomonas* dominated, which is inconsistent with the medium-temperature character of the Daqing oil reservoir.

### Survival of mesophilic non-spore-forming bacteria in autoclaved oil

We expected the active or germinated bacterial cells to be easily killed by autoclaving [26], which would have resulted in zero or negligible bacterial activity in crude oil degradation (Figure 1). Surprisingly, after inoculating the autoclaved crude oil with exogenous bacteria and incubating the cultures for 55 days, bacteria other than the inoculated ones were present and even dominated in the autoclaved crude oil cultures. Experimental contamination could be excluded because of two reasons: no bacteria could be detected in the three parallel controls and the bacteria detected in the inoculated cultures substantially differed from one another. Detection of *Bacillus* and *Paenibacillus* was not unexpected because they can form spores that are resistant to heat, desiccation, and many other extreme environmental stresses. However, the presence as well as dominance of non-spore-forming mesophilic bacteria, including *Pseudomonas, Dietzia, Alcaligenes*, and *Microbacterium* was surprising.

Many researchers have investigated the bacterial responses toward stress, including heat [27], [28], desiccation [26], [29], freezing and thawing [30], [31], chlorine disinfection [32], [33], and salt stress [34]. When encountering low water content, bacterial cells increase solute concentration in the cytoplasm by synthesizing organic solutes such as betaine [25], [35]. In addition, by selectively taking up inorganic solutes like K^+^ to lower water content [33] and by altering membrane structure [36], bacteria may develop increased heat resistance, in particular, if they are in a dormant state [25]. Compared with moist heat sterilization, dry heat sterilization requires higher temperatures and longer sterilization times to achieve the same level of disinfection [29]. In addition, air-pockets in autoclaves [37], sterilizing medium [38], use of sterilizing bags, and the manner in which bags are wrapped [39] influence disinfection efficiency. These investigations may explain the detection of non-spore-forming mesophilic bacteria in the crude oil culture. First, the bacteria may have been in a dormant state because of the extremely low nutrient and oxygen availability caused by the high hydrophobicity of crude oil. Second, the low water availability might also have increased the solute concentration in the cell cytoplasm, which may increase the heat resistance of the bacterial cells. Third, since the crude oil could not dissolve in the MSM, mixing of crude oil and water steam might not be complete, resulting in the formation of small crude oil drops. Bacterial cells inside crude oil drops may have avoided contact with steam, thereby gaining protection from steam autoclaving. Further studies are needed to investigate the exact mechanisms by which mesophilic non-spore-forming bacteria survive in autoclaved crude oil.

### Stimulation of the autoclaved crude oil bacterial community

In general, alkane-degrading bacteria can produce fatty acids through alkane hydroxylation [40], causing a decrease in the pH (Figure 1b). Some fatty acids are surface-active materials [41] and act as substrates for biosurfactant production, promoting the emulsification of crude oil and reducing the solution's surface tension (Figure 1c). The production of fatty acids and biosurfactants by some bacterial strains was reported to stimulate other bacteria, and even lead to turnover of the microbial community [42]–[44]. In addition, other intermediates can also stimulate different bacteria. For example, a non-benzo[a]pyrene-degrading *Rhodanobacter* strain could grow indirectly on benzo[a]pyrene after other consortium members produced metabolites from benzo[a]pyrene [45]. Therefore, if stimulated by fatty acids, biosurfactants, or other intermediates, the dormant bacteria capable of surviving autoclaving (e.g., *Pseudomonas* and *Bacillus*) may become active and show gradual growth.

Since different exogenous bacteria can produce different organic compounds, which include fatty acids and biosurfactants, they may stimulate the growth of different bacteria (Table 4, Figure 2). However, similar inoculants (e.g., bacteria in the same genus) had relatively similar stimulation patterns (Figure 3). For example, *Acinetobacter* spp. stimulated increased growth of *Bacillus*, while *Dietzia* spp. and *Pseudomonas* sp. stimulated increased growth of *Pseudomonas* (Table 4).

The fact that the bacterial clones retrieved from the original crude oil sample were rarely detected in the inoculated bacterial community, and vice versa, might be explained by the fact that different bacterial populations might be differently enriched during the incubation. Exogenous bacteria could have selectively stimulated the non-detectable bacteria in the original crude oil sample, which then made these bacteria detectable in the inoculated cultures. In addition, although the biomass was not detected, it is probable that the dominant bacterial population in the inoculated cultures could be much bigger than those in the original crude oil bacterial community. As a consequence, the small population in the original oil sample might not be easily detected.

It is noteworthy that although *Pseudomonas* could be detected in culture PA-13, they differed from the inoculated species. This result is consistent with the results obtained by Al-Hadhrami et al [46]; they demonstrated that bacteria stimulated in a nutrient-poor environment were more competitive than the phylogenetically similar, inoculated organisms that had been grown in a nutrient-rich medium. Moreover, because of the ease of stimulation and turnover in microbial communities, Head et al [47] suggested that the success of biodegradation is sometimes determined by the organisms that carry out important secondary functions and not by the inoculated microorganisms themselves.

In summary, mesophilic non-spore-forming bacteria in crude oil survived autoclaving, and could be differentially stimulated by different types of inoculant bacteria, but similarly by closely-related bacteria. This directed stimulation of endogenous bacteria could be useful in developing biological technologies for bioaugmentation and MEOR. Special efforts should be taken to evaluate the influence of endogenous bacteria capable of surviving autoclaving on the abilities of hydrocarbon-degrading bacteria, in particular, in relation to bioaugmentation and MEOR.

## Supporting Information

Figure S1 **Phylogenetic tree showing the genetic relationships among the clones retrieved from crude oil and the six cultures.** The tree was constructed by the neighbor-joining method using partial sequences of the 16S rRNA gene. Numbers of clones with identical sequences are shown in parentheses. The bar represents two substitutions per 100 nucleotide positions. Bootstrap probabilities of >70% are indicated at the branch nodes. The DDBJ/EMBL/GenBank accession numbers for reference strains and clones obtained in this study are shown in parentheses. The clones initiated with 1, 2, 6, 11, 13, and 24 represented the clones retrieved from AJ-1, DM-2, DP-6, AV-11, PA-13, and DM-24 cultures. The clones initiated with A and B represent those retrieved from crude oil. (a) Group AJ-1; (b) Group DM-2; (c) Group DP-6; (d) Group AV-11; (e) Group PA-13; (f) Group DM-24; and (g) Crude oil.
(DOC)

Figure S2 **Rarefaction curves of four bacterial 16S rRNA gene clone libraries.** OTUs were defined at a sequence similarity level of 97%. Curves were generated by 10,000 iterations of random sampling. Error bars represented 95% confidence intervals (CI) of the observed OTUs. (a) Group AJ-1; (b) Group DM-2; (c) Group DP-6; (d) Group AV-11; (e) Group PA-13; (f) Group DM-24; and (g) Crude oil.
(DOC)
