# Supplementary material for: Bacteria in Crude Oil Survived Autoclaving and Stimulated Differentially by Exogenous Bacteria
Source: PLoS One. 2012 Sep 17;7(9):e40842. doi: 10.1371/journal.pone.0040842 (PMC3444520; doi:10.1371/journal.pone.0040842)
Supplement: Figure S1 — Phylogenetic tree showing the genetic relationships among the clones retrieved from crude oil and the six cultures. The tree was constructed by the neighbor-joining method using partial sequences of the 16S rRNA gene. Numbers of clones with identical sequences are shown in parentheses. The bar represents two substitutions per 100 nucleotide positions. Bootstrap probabilities of >70% are indicated at the branch nodes. The DDBJ/EMBL/GenBank accession numbers for reference strains and clones obtained in this study are shown in parentheses. The clones initiated with 1, 2, 6, 11, 13, and 24 represented the clones retrieved from AJ-1, DM-2, DP-6, AV-11, PA-13, and DM-24 cultures. The clones initiated with A and B represent those retrieved from crude oil. (a) Group AJ-1; (b) Group DM-2; (c) Group DP-6; (d) Group AV-11; (e) Group PA-13; (f) Group DM-24; and (g) Crude oil. (DOC) [file pone.0040842.s001.doc]

**Supporting materials**

**Bacteria in crude oil survived autoclaving and were characteristically stimulated by different exogenous bacteria**

Xiao-Cui Gong*, Ze-Shen Liu*, Peng Guo, Chang-Qiao Chi, Jian Chen, Xing-Biao Wang, Yue-Qin Tang, and Xiao-Lei Wu

Department of Energy and Resources Engineering, College of Engineering, Peking University, Beijing 100871, P. R. China

* These authors contributed equally to this work

**Running title**: Crude oil bacteria survived autoclaving and stimulated

**Figure S1**

**(a)**

**(b)**

**(c)**

**(d)**

**(e)**

**(f)**

**(g)**
