# Supplementary material for: Bacteria in Crude Oil Survived Autoclaving and Stimulated Differentially by Exogenous Bacteria
Source: PLoS One. 2012 Sep 17;7(9):e40842. doi: 10.1371/journal.pone.0040842 (PMC3444520; doi:10.1371/journal.pone.0040842)
Supplement: Figure S2 — Rarefaction curves of four bacterial 16S rRNA gene clone libraries. OTUs were defined at a sequence similarity level of 97%. Curves were generated by 10,000 iterations of random sampling. Error bars represented 95% confidence intervals (CI) of the observed OTUs. (a) Group AJ-1; (b) Group DM-2; (c) Group DP-6; (d) Group AV-11; (e) Group PA-13; (f) Group DM-24; and (g) Crude oil. (DOC) [file pone.0040842.s002.doc]

**Supporting materials**

**Bacteria in crude oil survived autoclaving and were characteristically stimulated by different exogenous bacteria**

Xiao-Cui Gong*, Ze-Shen Liu*, Peng Guo, Chang-Qiao Chi, Jian Chen, Xing-Biao Wang, Yue-Qin Tang, and Xiao-Lei Wu

Department of Energy and Resources Engineering, College of Engineering, Peking University, Beijing 100871, P. R. China

* These authors contributed equally to this work

**Running title**: Crude oil bacteria survived autoclaving and stimulated

**Figure S2**

**(a)**

**(b)**

**(c)**

**(d)**

**(e)**

**(f)**

**(g)**
